# Genetic polymorphisms of autophagy-related gene 5 (*ATG5*) rs473543 predict different disease-free survivals of triple-negative breast cancer patients receiving anthracycline- and/or taxane-based adjuvant chemotherapy

**DOI:** 10.1186/s40880-018-0268-1

**Published:** 2018-01-31

**Authors:** Meiying Li, Fei Ma, Jiayu Wang, Qing Li, Pin Zhang, Peng Yuan, Yang Luo, Ruigang Cai, Ying Fan, Shanshan Chen, Qiao Li, Binghe Xu

**Affiliations:** Department of Medical Oncology, National Cancer Center/Cancer Hospital, Chinese Academy of Medical Sciences and Peking Union Medical College, No. 17, Panjiayuan Nanli, Chaoyang District, Beijing, 100021 P. R. China

**Keywords:** Autophagy-related gene 5, Triple-negative breast cancer, Disease-free survival, Anthracycline, Taxanes

## Abstract

**Background:**

Autophagy plays a crucial role in chemotherapy resistance of triple-negative breast cancer (TNBC). Hence, autophagy-related gene 5 (ATG5), an essential molecule involved in autophagy regulation, is presumably associated with recurrence of TNBC. This study was aimed to investigate the potential influence of single-nucleotide polymorphisms in *ATG5* on the disease-free survival (DFS) of early-stage TNBC patients treated with anthracycline- and/or taxane-based chemotherapy.

**Methods:**

We genotyped *ATG5* SNP rs473543 in a cohort of 316 TNBC patients treated with anthracycline- and/or taxane-based chemotherapy using the sequenom’s MassARRAY system. Kaplan–Meier survival analysis and Cox proportional hazard regression analysis were used to analyze the association between *ATG5* rs473543 genotypes and the clinical outcome of TNBC patients.

**Results:**

Three genotypes, AA, GA, and GG, were detected in the rs473543 of *ATG5* gene. The distribution of *ATG5* rs473543 genotypes was significantly different between patients with and without recurrence (*P* = 0.024). Kaplan–Meier survival analysis showed that patients carrying A allele of *ATG5* rs473543 had an increased risk of recurrence and shorter DFS compared with those carrying the variant genotype GG in rs473543 (*P* = 0.034). In addition, after adjusting for clinical factors, multivariate Cox regression analyses revealed that the AA/GA genotype of rs473543 was an independent predictor for DFS (hazard risk [HR], 1.73; 95% confidence interval [CI], 1.04–2.87; *P* = 0.034). In addition, DFS was shorter in node-negative patients with the presence of A allele (AA/GA) than in those with the absence of A allele (*P* = 0.027).

**Conclusion:**

*ATG5* rs473543 genotypes may serve as a potential marker for predicting recurrence of early-stage TNBC patients who received anthracycline-and/or taxane-based regimens as adjuvant chemotherapy.

## Background

Triple-negative breast cancer (TNBC), characterized by absent or minimal expression of hormone receptor [estrogen receptor (ER) and progesterone receptor (PR)] and human epidermal growth factor receptor 2 (HER2), constitutes 10%–20% of all breast cancers [1–3]. TNBC occurs more frequently in young patients and generally behaves aggressively, with early distant metastases and consequently poor prognosis [4, 5]. Because of lack of available targeted or biological agents, chemotherapy is the mainstay treatment of TNBC, and anthracyclines and taxanes remain the standard of care for moderate-to-high-risk TNBC in the adjuvant setting [6]. Although initially responsive to chemotherapy, a high proportion of TNBC patients eventually develop resistance, resulting in treatment failure and recurrence [7, 8]. Since the vast majority of cancer deaths are related to disseminated diseases, novel molecular prognostic markers able to predict resistance to chemotherapeutic agents and metastatic risk in TNBC patients would be extremely valuable.

Autophagy is an evolutionarily conserved catabolic process that is primarily responsible for the removal and recycling of long-lived proteins and damaged organelles to maintain the homeostasis of the cell [9, 10]. It is mainly activated by stress and nutrient deprivation and occurs in both normal and cancer cells [11]. Recently, increasing evidence indicates that autophagy has a cytoprotective function enabling cancer cells to cope with cytotoxic or other stresses induced by chemotherapy [12]. Importantly, it has been reported that inhibiting autophagy could augment the anticancer efficacy of epirubicin on both anthracycline-sensitive and -resistant TNBC [13]. Meanwhile, Wen et al. [12] demonstrated that autophagy inhibition could re-sensitize paclitaxel-resistant TNBC cells to paclitaxel-induced apoptosis. Accordingly, it is acknowledged that autophagy plays a crucial role in the resistance of TNBC cells to anthracyclines and taxanes. Therefore, we deduced that autophagy may be closely related with the prognosis of TNBC patients who were treated with anthracycline- and/or taxane-based adjuvant chemotherapy. Given autophagy is a highly regulated process involving a series of key molecules [14], the relationship between these molecules and the disease-free survival (DFS) of TNBC patients deserves further investigation.

Among all the molecular regulators, autophagy-related gene 5 (ATG5) is an E3 ubiquitin ligase essential for autophagy due to its role in autophagosome elongation [15]. Results from previous researches showed that altered *ATG5* expression and/or selective allelic loss of *ATG5* are associated with malignancy development, treatment resistance, and tumor progression [16–20]. These findings indicate that ATG5 may serve as a novel predictor for the prognosis of cancer patients. In fact, overexpression of ATG5 has been recently reported as a novel predictor for favorable DFS in breast cancer patients [21]. As single-nucleotide polymorphism (SNP) could contribute to the altered gene expression [22, 23], it is highly possible that specific SNPs in critical genes may have potential influence on the disease outcomes of patients with breast cancer. However, the clinical significance of *ATG5* SNPs in TNBC patients has never been elucidated.

In the present study, we genotyped the *ATG5* SNP rs473543 in a cohort of Chinese women with early-stage TNBC who received adjuvant chemotherapy with anthracyclines and/or taxanes to explore its role as a predictor of the clinical outcome of these patients.

## Materials and methods

### Patients and blood samples

We reviewed the electronic records of breast cancer patients treated at Cancer Hospital, Chinese Academy of Medical Sciences (CAMS) between November 1999 and June 2015. The patient selection criteria were as follows: (1) all the patients were female; (2) each patient had complete clinicopathological data, including patient’s age, tumor size, axillary lymph node status, TNM stage, pathological type, vascular invasion, adjuvant chemotherapy and radiotherapy; (3) the patient was pathologically diagnosed with TNBC; (4) the patient was diagnosed with stage I–III TNBC; and (5) the patient had received anthracycline- and/or taxane-containing regimens as adjuvant chemotherapy. The blood samples of the selected patients were derived from the sample bank which has been built to collect tumor tissues and blood samples from breast cancer patients who were treated in our hospital since 1998. Patients without complete clinical information and sufficient blood samples were excluded.

### Breast cancer subtype definition

Estrogen receptor and PR statuses were evaluated based on the immunohistochemical (IHC) results of formalin-fixed, paraffin-embedded, primary breast cancer tissues obtained from patients. ER-positive and PR-positive statuses are defined by ≥ 1% nuclear staining. IHC and/or fluorescence in situ hybridization (FISH) were routinely conducted to determine the HER2 status. Breast cancers are classified as HER2-positive if they are scored as 3+ with uniform membrane staining for HER2 in ≥ 10% tumor cells demonstrated by IHC or *HER2* gene amplification demonstrated by FISH [single-probe, average *HER2* copy number ≥ 6 signals/cell; dual-probe *HER2/*chromosome 17 centromere (*CEP17*) ratio ≥ 2.0 with an average *HER2* copy number ≥ 4 signals/cell; dual-probe *HER2/*chromosome enumeration ratio ≥ 2.0 with an average *HER2* copy number < 4 signals/cell; *HER2/CEP17* ratio < 2.0 with an average *HER2* copy number ≥ 6 signals/cell]. Tumors negative for ER, PR, and HER2 were defined as TNBC.

### Treatment

The EC regimen [epirubicin (EPI) 90 mg/m^2^ or pirarubicin (THP) 40–50 mg/m^2^ on day 1 and cyclophosphamide (CTX) 600 mg/m^2^ on day 1, repeated every 21 days for 4 cycles], EC-T regimen [EPI 90 mg/m^2^ and CTX 600 mg/m^2^ on day 1, repeated every 14 or 21 days for 4 cycles, followed by docetaxel (DOC) 80 mg/m^2^ on day 1, repeated every 21 days for 4 cycles or paclitaxel (TAX) 175 mg/m^2^ on day 1, repeated every 14 or 21 days for 4 cycles], ET regimen (EPI 75 mg/m^2^ or THP 40–50 mg/m^2^ on day 1 and DOC 75 mg/m^2^ or TAX 175 mg/m^2^ on day 2, repeated every 21 days for 6 cycles), TAC regimen (EPI 75 mg/m^2^ or THP 40–50 mg/m^2^, CTX 500 mg/m^2^, and TAX 175 mg/m^2^ or DOC 75 mg/m^2^ on day 1, repeated every 21 days for 6 cycles), and CAF regimen [CTX 500 mg/m^2^ on day 1, EPI 75 mg/m^2^ or THP 40–50 mg/m^2^ or doxorubicin (ADM) 50 mg/m^2^ on day 1, 5-fluorouracil [5-FU] 500 mg/m^2^ on days 1 and 8, repeated every 21 days for 6 cycles] were classified as anthracycline-based regimens; the EC-T regimen, TAC regimen, ET regimen, TC regimen (DOC 75 mg/m^2^ or TAX 175 mg/m^2^ and CTX 600 mg/m^2^ on day 1, repeated every 21 days for 4 cycles) and carboplatin-taxane regimen [DOC 75 mg/m^2^ or TAX 175 mg/m^2^ on day 1, and carboplatin (CAPE) AUC = 5 mg/mL on day 2, repeated every 21 days for 6 cycles] were classified as taxane-based regimens; the EC-T regimen, ET regimen, and TAC regimen were classified as anthracycline-taxane combinational regimens.

### Follow-up

Patients were followed up every 3 months during the first year after surgery, then every 4 months in the second year and every 6 months in years 3–5. After that, patients were followed annually until February 17, 2017. Disease progression was diagnosed based on imaging results [computed tomography (CT), magnetic resonance imaging, or positron emission tomography/computed tomography (PET/CT)] and/or biopsy of the metastatic lesions. DFS was defined as the duration between the date of surgery and the date of the first event (locoregional recurrence or distant metastasis or death from any cause, whichever occurred first). Patients who were recurrence-free and alive at the last follow-up were censored.

### Selection of tag SNP in the *ATG5* gene

First, we screened the National Center for Biotechnology Information (NCBI) SNP database and selected SNPs located in the promoter region, exon, 5′-untranslated region (UTR), and 3′-UTR of the *ATG5* gene. The minor allele frequency of the selected SNPs should be more than 0.05. Second, we searched the PubMed database for articles reporting significant roles of candidate *ATG5* SNPs in the development, progression, and chemotherapy resistance of different cancers. Combining these research results, we finally decided to genotype four *ATG5* SNPs of potential interest (rs473543, rs28656919, rs3761796, and rs506027).

### DNA preparation and genotyping

Genomic DNA was isolated from the whole blood using the blood DNA kit (BioTeKe Corpration, Beijing, China) according to the manufacturer’s protocols. Genotyping was performed with the MassARRAY MALDI-TOF System (Sequenom Inc., San Diego, CA, USA). Primers (forward 5′-ACGTTGGATGAGGTGAAAGGTGATTACTTG-3′ and reverse 5′-ACGTTGGATGGGAAGAGAGAAGGACAAGGG-3′) for polymerase chain reaction or single-base extension were designed using the Assay Designer’s software version 3.0 (Sequenom Inc.) and synthesized by the Beijing Genomics Institute (Beijing, China).

Purified primer extension reaction products were dispensed onto a 384-well Spectro CHIP bioarray using MassARRAY Nanodispenser RS1000 (Sequenom Inc.) and determined by the matrix-assisted laser desorption/ionization time-off light mass spectrometer. Genotype analysis was performed through the MassARRAY Typer software version 4.0 (Sequenom Inc.). Negative controls (without DNA) and duplicate samples were included for quality assurance of genotyping.

### Statistical analysis

Chi square test was used to evaluate the Hardy–Weinberg equilibrium. SNPs that were not in Hardy–Weinberg equilibrium were excluded from analysis. The survival probability was calculated using the Kaplan–Meier method. Differences across survival curves were compared by the log-rank test. Using Chi square test, we compared the differences in clinicopathological characteristics between patients with and without recurrence. Univariate and multivariate survival analyses were performed using the Cox proportional hazard regression model. The following variables were examined in the univariate analyses for their relations with DFS: patients’ age, tumor size, axillary lymph node status, TNM stage, pathological type, vascular invasion, adjuvant radiotherapy, and *ATG5* rs473543 genotype. Factors with a univariate relevant influence on DFS were then included in the multivariate survival analyses. The statistical analyses were performed with the software package SPSS 17.0 (SPSS, Inc., Chicago, IL, USA). A *P* value of less than 0.05 was considered statistically significant.

## Results

### Patient characteristics

A total of 316 TNBC patients were selected. All patients were Han Chinese. The median age of patients was 48 years (range 24–76 years). Of the 316 TNBC patients, 28 (8.9%) were treated with the EC regimen; 64 (20.3%) with the EC-T regimen; 14 (4.4%) with the TAC regimen; 74 (23.4%) with the ET regimen; 20 (6.3%) with the TC regimen; 75 (23.7%) with the carboplatin-taxane combination; 21 (6.6%) with the CAF regimen; 20 (6.3%) with other anthracycline- or taxane-based regimens, without information on doses of chemotherapeutic agents.

Among the four *ATG5* SNPs of potential interest (rs473543, rs28656919, rs3761796, and rs506027), rs473543 was in Hardy–Weinberg equilibrium. Details of the clinicopathological characteristics according to *ATG5* rs473543 genotypes are summarized, and no significant associations were observed between *ATG5* rs473543 genotypes and the clinicopathological characteristics (Table 1).

**Table 1:** Clinicopathological characteristics of patients with triple-negative breast cancer (TNBC) with respect to autophagy-related gene 5 (*ATG5*) rs473543 genotypes

Characteristic	Total [cases (%)]	*ATG5* rs473543 genotype [cases (%)]			*P*
		AA	GG	GA	
Total	316	60	112	144	
Age (years)					0.233
≤ 40	74 (23.4)	11 (18.3)	23 (20.5)	40 (27.8)	
> 40	242 (76.6)	49 (81.7)	89 (79.5)	104 (72.2)	
T stage					0.231
pT0–T1	156 (49.4)	35 (58.3)	50 (44.6)	71 (49.3)	
pT2–T4	160 (50.6)	25 (41.7)	62 (55.4)	73 (50.7)	
Axillary lymph node status					0.543
Negative	199 (63.0)	41 (68.3)	67 (59.8)	91 (63.2)	
Positive	117 (37.0)	19 (31.7)	45 (40.2)	53 (36.8)	
TNM stage					0.948
0–I	109 (34.5)	20 (33.3)	38 (33.9)	51 (35.4)	
II–III	207 (65.5)	40 (66.7)	74 (66.1)	93 (64.6)	
Pathological type					0.855
Invasive ductal carcinoma	294 (93.0)	58 (96.7)	102 (91.1)	134 (93.1)	
Grade 1/2	109 (34.5)	24 (40.0)	37 (33.0)	48 (33.3)	
Grade 3	164 (51.9)	30 (50.0)	57 (50.9)	77 (53.5)	
Unknown	21 (6.6)	4 (6.7)	8 (7.1)	9 (6.3)	
Others	22 (7.0)	2 (3.3)	10 (8.9)	10 (6.9)	
Vascular invasion					0.594
Yes	46 (14.6)	7 (11.7)	15 (13.4)	24 (16.7)	
No	270 (85.4)	53 (88.3)	97 (86.6)	120 (83.3)	
Adjuvant radiotherapy					0.396
Yes	121 (38.3)	21 (35.0)	39 (34.8)	61 (42.4)	
No	195 (61.7)	39 (65.0)	73 (65.2)	83 (57.6)	
Chemotherapeutic regimens^a^					0.455
Anthracycline-based	222 (70.3)	43 (71.7)	67 (59.8)	112 (77.8)	
Taxane-based	251 (79.4)	47 (78.3)	95 (84.8)	109 (75.7)	
Anthracycline-taxane combination	157 (70.7)	30 (50.0)	50 (44.6)	77 (53.5)	

^a^Patients who received anthracyclines as chemotherapeutic treatment may also receive taxanes as chemotherapy at the same time. Double counting resulted in the sum higher than the number of subjects investigated in this study

### Association between *ATG5* rs473543 genotypes and DFS

The median follow-up duration was 66.7 months (range 2.8–206.3 months). By the end of follow-up, 81 patients (25.6%) had recurrence. Besides, 58 (71.6%) patients in the recurrent group had locoregional recurrence or distant metastasis within 3 years after adjuvant chemotherapy.

Using Chi square test, we found that axillary lymph node status, TNM stage, and vascular invasion were significantly different between the recurrent and non-recurrent groups (Table 2). Meanwhile, the distribution of *ATG5* rs473543 genotypes was also significantly different between these two groups, suggesting that *ATG5* rs473543 genotypes were related to the prognosis of early-stage TNBC patients (Table 2).

**Table 2:** Different clinicopathological characteristics in the recurrent group and the non-recurrent group of TNBC patients

Characteristic	Non-recurrent group [cases (%)]	Recurrent group [cases (%)]	*P* value
Total	235	81	
Age (years)			0.768
≤ 40	56 (23.8)	18 (6.7)	
> 40	179 (76.2)	63 (77.8)	
Family history of breast cancer			0.396
Yes	35 (14.9)	9 (11.1)	
No	200 (85.1)	72 (88.9)	
T stage			0.199
pT0–T1	121 (51.5)	35 (43.2)	
pT2–T4	114 (48.5)	46 (56.8)	
Axillary lymph node status			< 0.001
Negative	163 (69.4)	36 (44.4)	
Positive	72 (30.6)	45 (55.6)	
TNM stage			< 0.001
0–I	91 (38.7)	18 (22.2)	
II–III	144 (61.3)	63 (77.8)	
Pathological type			0.407
Invasive ductal carcinoma	217 (92.3)	77 (95.0)	
Grade 1/2	75 (31.9)	34 (42.0)	
Grade 3	131 (55.7)	33 (40.7)	
Unknown	11 (4.7)	10 (12.3)	
Others	18 (7.7)	4 (5.0)	
Vascular invasion			0.003
Yes	26 (11.1)	20 (24.7)	
No	209 (88.9)	61 (75.3)	
Adjuvant radiotherapy			0.429
Yes	87 (37.0)	34 (42.0)	
No	148 (63.0)	47 (58.0)	
*ATG5* rs473543 genotype			0.024
AA	46 (19.6)	14 (17.3)	
GA	97 (41.3)	47 (58.0)	
GG	92 (39.1)	20 (24.7)	

**Fig. 1:**
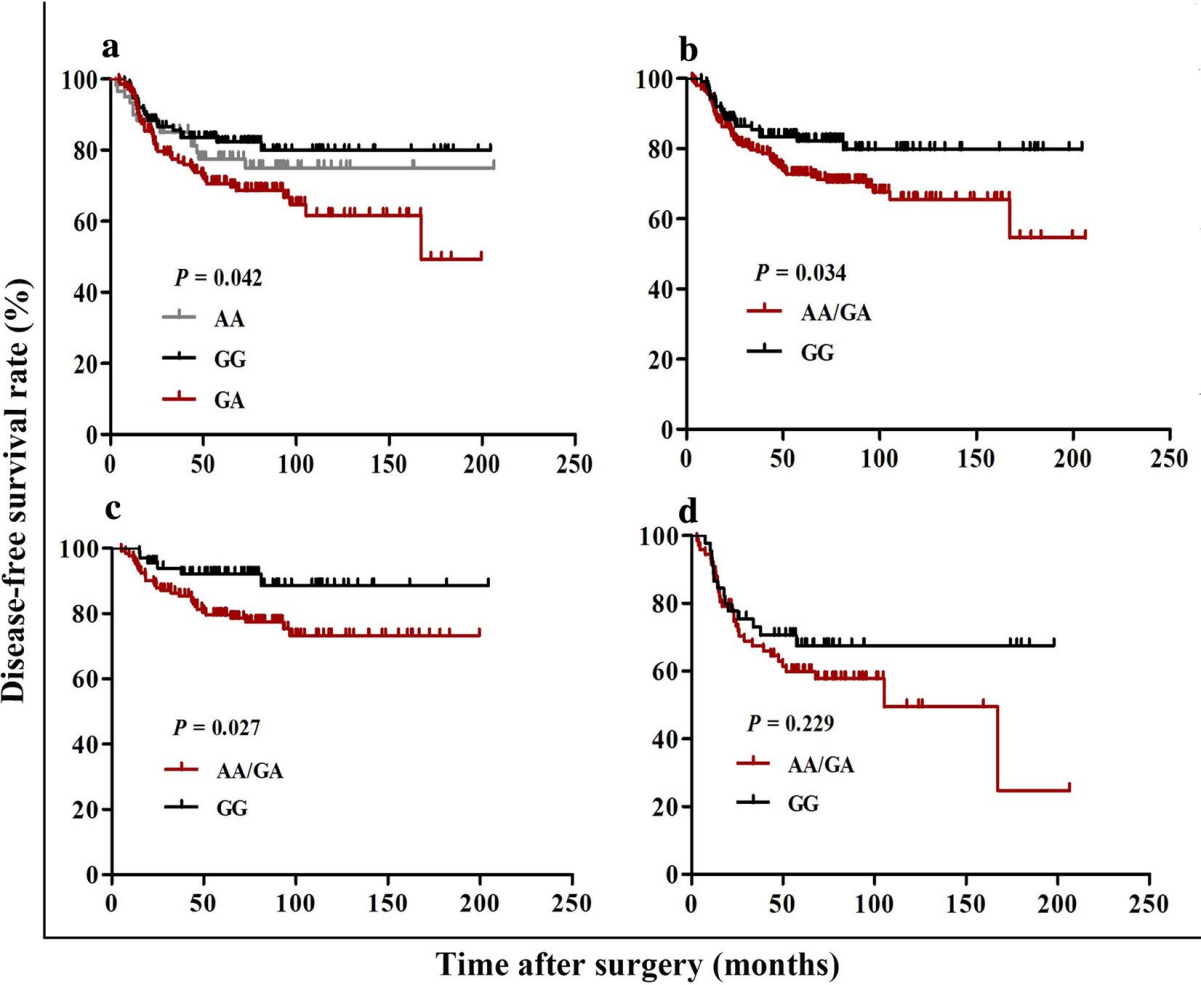
Association between the autophagy-related gene 5 (*ATG5*) single-nucleotide polymorphism (SNP) rs473543 genotypes and disease-free survival (DFS) in early-stage triple-negative breast cancer (TNBC) patients. DFS of the patients grouped according to *ATG5* rs473543 genotypes. **a** GG vs. GA vs. AA in the whole cohort; **b** GG vs. AA/GA in the whole cohort; **c** GG vs. AA/GA among patients without axillary lymph node involvement; **d** GG vs. AA/GA among patients with axillary lymph node involvement

Kaplan–Meier curves for DFS show that *ATG5* rs473543 genotypes were significantly associated with DFS in TNBC patients (*P* = 0.042; Fig. 1a). What’s more, patients with the AA/GA genotype had an increased risk of recurrence and shorter DFS than patients with the GG genotype (*P* = 0.034; Fig. 1b).

In addition, we performed stratified analyses by the clinical characteristics including age, family history of breast cancer, axillary lymph node status, TNM stage, tumor grade, and vascular invasion to further clarify the prognostic value of *ATG5* rs473543 genotypes in TNBC patients. Using Kaplan–Meier analysis, we found that in node-negative patients, the presence of A allele (AA/GA) was associated with shorter DFS than the absence (*P* = 0.027; Fig. 1c). However, no significant associations between *ATG5* rs473543 genotypes and DFS were seen in node-positive patients (*P* = 0.229; Fig. 1d) and other clinical subgroups (Table 3).

**Table 3:** The relationship between *ATG5* rs473543 genotypes and DFS in the recurrent group and the non-recurrent group of TNBC patients

Characteristic	Non-recurrent group (cases)		Recurrent group (cases)		*P* value
	AA/GA	GG	AA/GA	GG	
Total	143	92	61	20	
Age (years)					
≤ 40	36	20	15	3	0.139
> 40	107	72	46	17	0.093
Family history of breast cancer					
Yes	18	17	8	1	0.081
No	125	75	53	19	0.122
Axillary lymph node status					
Negative	102	61	30	6	0.027
Positive	41	31	31	14	0.229
TNM stage					
0–I	56	35	15	3	0.097
II–III	87	57	46	17	0.127
Invasive ductal carcinoma					
Grade 1/2	47	28	25	9	0.410
Grade 3	82	49	25	8	0.179
Vascular invasion					
Yes	16	10	15	5	0.444
No	127	82	46	15	0.068

*DFS* disease-free survival

In different subclinical groups, the relationship between *ATG5* rs473543 genotypes and DFS was analyzed using Kaplan–Meier curves. Differences across survival curves were compared by the log-rank test, the *P* values for which were shown in this table

### Univariate and multivariate analyses of DFS

Univariate analysis of DFS demonstrated that axillary lymph node metastases (hazard risk [HR], 2.507; 95% confidence interval [CI], 1.615–3.890; *P* < 0.001), late TNM stage (HR 2.063; 95% CI 1.221–3.485; *P* = 0.007), vascular invasion (HR 2.486; 95% CI 1.497–4.126; *P* < 0.001), and the AA/GA genotype of *ATG5* rs473543 (HR 1.717; 95% CI 1.036–2.847; *P* = 0.036) were significantly associated with a high risk of TNBC recurrence (Table 4).

**Table 4:** Univariate and multivariate analyses of prognostic factors for DFS of TNBC patients

Characteristic	Univariate analysis		Multivariate analysis	
	HR (95% CI)	*P* value	HR (95% CI)	*P* value
Age (≤ 40 vs. > 40 years)	1.038 (0.614–1.752)	0.890	–	
T stage (pT0–T1 vs. pT2-T4)	1.393 (0.897–2.164)	0.140	–	
Family history of breast cancer (yes vs. no)	0.771 (0.386–1.543)	0.463	–	
Axillary lymph node status (negative vs. positive)	2.507 (1.615–3.890)	< 0.001	2.055 (1.190–3.547)	0.010
TNM stage (0-I vs. II-III)	2.063 (1.221–3.485)	0.007	1.237 (0.653–2.346)	0.514
Pathological type (invasive ductal carcinoma vs. others)	0.874 (0.450–1.695)	0.689	–	
Vascular invasion (yes vs. no)	2.486 (1.497–4.126)	< 0.001	1.901 (1.124–3.216)	0.017
*ATG5* rs473543 genotype (AA/GA vs. GG)	1.717 (1.036–2.847)	0.036	1.729 (1.041–2.870)	0.034

*HR* hazard ratio, *CI* confidence interval, *–* not included

The results of multivariate Cox regression analysis validated axillary lymph node metastases (HR 2.055; 95% CI 1.190–3.547; *P* = 0.010), vascular invasion (HR 1.901; 95% CI 1.124–3.216; *P* = 0.017), and *ATG5* rs473543 genotypes (HR 1.729; 95% CI 1.041–2.870; *P* = 0.034) as independent prognostic factors for DFS (Table 4).

## Discussion

In the present study, we focused on the association between *ATG5* rs473543 genotypes and the prognosis of TNBC patients who were treated with adjuvant anthracycline- and/or taxane-based regimens. No significant association was discovered between clinicopathological characteristics and *ATG5* rs473543 genotypes. The Kaplan–Meier survival analysis revealed that compared with the GG genotype, the AA/GA genotype was significantly associated with increased risk of recurrence and thus unfavorable disease outcomes. Furthermore, univariate and multivariate Cox regression analyses confirmed that ATG5 rs473543 genotypes were an independent predictor for DFS. Besides, a stratified analysis according to patients’ lymph node status showed that in patients without axillary lymph node metastasis, the rs473543 AA/GA genotype indicated a higher risk of recurrence and shorter DFS as compared with the GG genotype.

Recently, a growing body of researches has demonstrated that heightened autophagy is a mechanism of resistance for cancer cells faced with therapeutic stress [24–26]. Meanwhile, ATG5 is a key player and its des-regulation is closely related to chemoresistance in a variety of cancers. In gastric cancer patients who received epirubicin, cisplatin, and 5-FU adjuvant chemotherapy, up-regulated expression of ATG5 was identified as an important molecular feature of chemoresistance [18]. Consistent with this study, a recently published paper reported that down-regulating ATG5 could enhance 5-FU-induced autophagy-associated and apoptosis-independent cell death in esophageal cancer cells [27]. Meanwhile, Chittaranjan et al. [13] have demonstrated that the deletion of *ATG5* could augment the anticancer effects of EPI on both anthracycline-sensitive and -resistant TNBC. On the contrary, knockdown of *ATG5* in osteosarcoma cells has an opposing effect on camptothecin-induced cytotoxicity [19]. In addition, some studies demonstrated that elevated expression of ATG5 was associated with favorable clinical outcomes in both breast cancer [21] and melanoma patients [28]. Taken together, the expression level of ATG5 may serve as a valuable indicator for chemoresistance and prognosis of cancer patients. However, the influence of *ATG5* genotypes on cancer prognosis remains largely unexplored, and only one study suggested that heterozygous loss of *ATG5* was associated with resistance to anticancer treatment and metastasis risk in melanoma patients [17].

The present study demonstrated that the *ATG5* rs473543 AA/GA genotype was an independent predictor of short DFS in TNBC patients. The frequency of the AA/GA genotype was 64.6%, which means a large proportion of patients were at a high risk of recurrence because of carrying the A allele. Although the frequency of the AA/GA genotype in the whole Chinese population was unknown, the prognostic value of rs4735431 genotyes should not be neglected. Moreover, all patients in the present study received anthracycline- and/or taxane-based regimens as adjuvant treatment, which are the most commonly used protocols for treating TNBC in real world. Therefore, our data indicated that the *ATG5* rs473543 may serve as a potential prognostic biomarker in TNBC patients receiving anthracycline- and/or taxane-based regimens as adjuvant therapy.

Subgroup analysis indicated that in patients without axillary lymph node metastasis, the *ATG5* rs473543 AA/GA genotype was significantly related with short DFS, suggesting that treatment regimens could be optimized according to the *ATG5* rs473543 genotype to improve the clinical outcomes in lymph node-negative TNBC patients. No significant associations between *ATG5* rs473543 genotypes and disease progression were seen in other clinical subgroups. More randomized, prospective researches are needed to elucidate whether *ATG5* rs473543 genotypes could predict the recurrence of TNBC patients with specific clinical features.

All the results mentioned above are consistent with previous reports describing the great clinical importance of ATG5 in cancers. Nonetheless, the underlying molecular mechanisms of the prognostic importance of *ATG5* rs473543 genotypes in TNBC patients are not known. We learned from prior researches that promoter SNPs of genes could lead to aberrant gene expression, thus conferring increased risk of cancer development as well as influence on the prognoses of cancer patients [29, 30]. In the present study, the variant rs473543 is located within the promoter of *ATG5*, which may in part explain its significance in predicting the prognosis of TNBC patients. However, whether *ATG5* rs473543 genotypes affect the DFS of early-stage TNBC patients through regulating ATG5 expression warrants further investigation.

Despite the aforementioned evidence, we also acknowledge some limitations of the present study. First, the cohort was of a moderate sample size, which may have led to limited statistical power. Hence, additional studies with larger independent populations are needed to further clarify the association between *ATG5* rs473543 genotypes and the clinical outcomes in TNBC patients. Second, the mechanisms of the effect of *ATG5* rs473543 genotypes were not investigated on cellular and molecular levels. Further researches concerning whether *ATG5* rs473543 genotypes could influence the prognosis of TNBC patients through transcription, mRNA stabilization, or post-translational regulation of ATG5 expression are urgently needed.

## Conclusions

The present study demonstrated that the SNP rs473543 in *ATG5* was associated with DFS and recurrence risk in early-stage TNBC patients who received anthracycline- and/or taxane-based regimens as adjuvant chemotherapy. In multivariate analyses, the *ATG5* rs473543 genotype emerges as a promising predictor for the clinical outcomes in TNBC patients. Besides, *ATG5* rs473543 genotypes could be used to optimize adjuvant regimens of lymph node-negative TNBC patients. Future prospective studies with larger sample sizes are warranted to confirm our findings.
